# The Nucleotide Capture Region of Alpha Hemolysin: Insights into Nanopore Design for DNA Sequencing from Molecular Dynamics Simulations

**DOI:** 10.3390/nano5010144

**Published:** 2015-01-27

**Authors:** Richard M. A. Manara, Susana Tomasio, Syma Khalid

**Affiliations:** School of Chemistry, University of Southampton, Highfield Campus, Southampton SO17 1BJ, UK; E-Mails: rm16g09@soton.ac.uk (R.M.A.M.); susana@cresset-group.com (S.T.)

**Keywords:** alpha-hemolysin, exonuclease sequencing, molecular dynamics, nanopore sequencing

## Abstract

Nanopore technology for DNA sequencing is constantly being refined and improved. In strand sequencing a single strand of DNA is fed through a nanopore and subsequent fluctuations in the current are measured. A major hurdle is that the DNA is translocated through the pore at a rate that is too fast for the current measurement systems. An alternative approach is “exonuclease sequencing”, in which an exonuclease is attached to the nanopore that is able to process the strand, cleaving off one base at a time. The bases then flow through the nanopore and the current is measured. This method has the advantage of potentially solving the translocation rate problem, as the speed is controlled by the exonuclease. Here we consider the practical details of exonuclease attachment to the protein alpha hemolysin. We employ molecular dynamics simulations to determine the ideal (a) distance from alpha-hemolysin, and (b) the orientation of the monophosphate nucleotides upon release from the exonuclease such that they will enter the protein. Our results indicate an almost linear decrease in the probability of entry into the protein with increasing distance of nucleotide release. The nucleotide orientation is less significant for entry into the protein.

## 1. Introduction

DNA sequencing using nanopores is a method that allows for direct analysis of genomic DNA [1,2,3,4,5,6,7,8]. Benefits of nanopore-based sequencing include detection of epigenetic markers [9,10], which is not possible using chemical methods as well as reducing costs compared to more traditional methods. The early promise has been realized by Oxford Nanopore Technologies in 2011–2012, however, the initial devices continue to be upgraded and improved to push the boundaries of what this technology can achieve.

**Figure 1:**
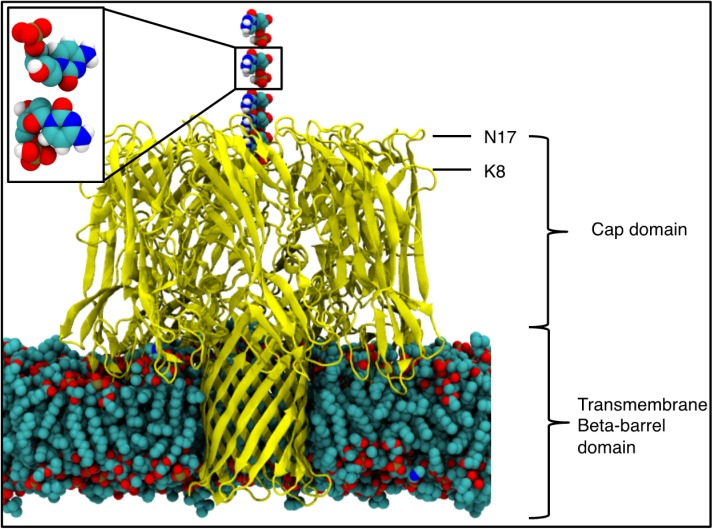
The alpha-hemolysin (αHL) protein in a 1,2-dimyristoyl-sn-glycero-3-phosphocholine (DMPC) bilayer, with the mononucleotide positioned above the vestibule entrance, where each mononucleotide represents an individual simulation. The protein is represented by yellow ribbons and for other atoms: Carbon is shown in cyan, oxygen in red, nitrogen in blue, phosphorus in brown and hydrogen in white. The waters and ions are excluded for clarity. (**Inset**) The phosphate orientations used, termed the “up” and “down” orientations, shown top and bottom respectively.

The pore forming toxin, alpha-hemolysin (αHL) from *S. aureus* [11] is perhaps the best studied example of a proteinaceous nanopore for DNA sequencing [12]. This protein has two domains, the vestibule or cap domain and the transmembrane pore domain (Figure 1) [11]. While αHL is a robust protein that is resistant to changes in pH and temperature over practical ranges, issues related to the high voltage thresholds required for DNA translocation render wild type αHL unsuitable for incorporation into a sequencing device. However, engineered mutants have shown considerable success for DNA sequencing, although they too are continuously being improved for more efficient DNA sequencing. For example, methods to improve the accuracy of the device by decreasing the rate of translocation of ssDNA [2,13] through the protein pores are being investigated. It has been proposed that incorporation of an exonuclease enzyme, which would split the ssDNA into individual mononucleotides before they enter the nanopore, may be an efficient way to reduce the translocation rate [10,14]. Selection of an exonuclease enzyme for incorporation into an alpha-hemolysin based sequencing device is dependent upon practical considerations such as temperature and pH dependence, size and conformational flexibility of the enzyme. Furthermore it is imperative that the enzyme is positioned relative to the αHL such that there is a practical level of confidence mononucleotides exiting the enzyme will enter the αHL and not simply diffuse away in solution. This information is vital for the practical design and construction of the chimera protein.

Molecular dynamics simulations provide a route to study the translocation of biological molecules through nanoscale pores at levels of detail that are difficult to achieve with experimental methods alone [15,16,17,18,19]. Here we employ atomistic molecular dynamics simulations to characterize the “nucleotide capture area” of the wildtype alpha-hemolysin protein. Capture of the cytosine monophosphate (CMP), was investigated by embedding alpha-hemolysin in a 1,2-dimyristoyl-sn-glycero-3-phosphocholine (DMPC) bilayer and solvating in 1 M NaCl, resulting in a simulation system composed of 273,000 atoms. Only one of the four mononucleotides was simulated due to the similarity in their masses with respect to the protein. The distance of CMP from the protein was varied. Specifically, CMP was positioned above the protein on the vestibule side such that it was located centrally, above the entrance to the protein (where central is defined here as the average co-ordinates of the C-alpha atoms of K8 residues which are in the vestibule domain of the protein) as shown in Figure 1. Simulations were performed with the nucleotide initially positioned at 10, 15, 20, 30 and 40 angstroms above the ring of K8 residues, along the principal axis of the protein. Additional systems were set up in which, at a distance of 30 angstroms from the K8 residues, the nucleotides were displaced in the plane parallel to the membrane, by 5, 10, 15 and 20 angstroms. Two sets of simulations were performed for each location of CMP, in which the CMP was orientated with either the phosphate moiety furthest or closest to the vestibule entrance, which we define as phosphate “up” or “down” orientations respectively, shown in the inset of Figure 1. For each location, 20 independent simulations of 5 ns duration were performed at 310 K, with an applied electric field of magnitude 0.1 V·nm^−1^ equivalent to approximately 350 mV across the membrane. Simulations were performed using the GROMACS [20] software package, version 4.5.5 [20,21] with the GROMOS53a6 [22] forcefield and the SPC model of water [23]. Further details of the methodology are provided in the Experimental Section.

## 2. Results and Discussion

For the purposes of clarity when reporting our results, we use two terms for the behaviour of the nucleotide with respect to the protein: “capture” and “possible capture”. These are defined as follows: When the entire CMP is below the ring of C-alpha atoms of N17, it is regarded as captured as over all simulations we observed no examples of exit from the vestibule entrance once interacting with the protein below this region. In contrast, if the CMP is entirely above this ring, it is deemed not captured. The final alternative is when simultaneously parts of the CMP are above and below the N17 ring, which we refer to as possible capture, as it was observed that CMP in this region, which we describe as the “edge” of the vestibule entrance, is capable of either entering the vestibule or diffusing into solution.

**Figure 2:**
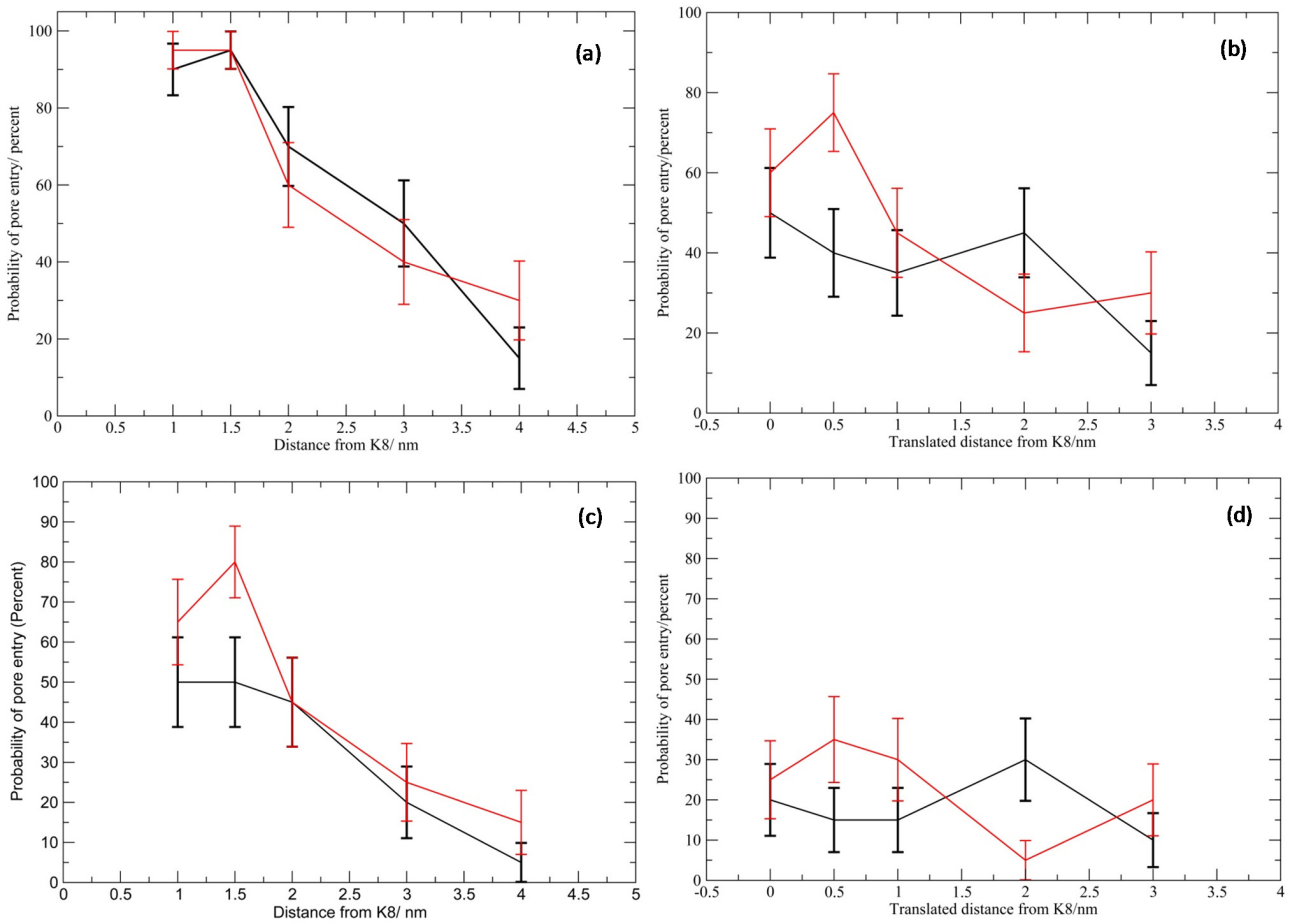
Plots showing the relaionship between release loction and probability of capture. The highest possible probability of entry for (**a**) the distance study and (**b**) the displacement study, e.g., these are the simulations where either capture or possible capture is observed. The lowest possible probability of entry for (**c**) the distance study and (**d**) the displacement study, e.g., these are the simulations where capture is observed. The black lines are for the phosphate up orientation and the red lines are the phosphate down orientation. Error bars were calculated by using the standard error, treating the data as a binomial distribution.

As the distance from which the mononucleotide is released from the protein is increased, the probability of entry into the vestibule decreases almost linearly, this reduction in the likelihood of entry occurs at approximately the same rate regardless of phosphate orientation (Figure 2). The probability of capture is higher with the phosphate in the down orientation, by an average of 10%. The phosphate orientation has negligible impact on the probability of “possible capture”.

As the CMP is displaced away from the centre of the vestibule entrance at a distance of 30 angstroms, in general, the probability of entry decreases, however we note that there is an increased probability of entry with translation of 0.5 nm (Figure 2). We theorize that this is due to the CMP now being released above the edge of the vestibule entrance instead of above the centre; therefore, less lateral diffusion is required to interact with the protein. Here we note a higher correlation between the phosphate orientation and entry, with the phosphate down orientation demonstrating an average of 15% higher entry probability. The minimum probability of entry is generally higher by 10% with the CMP in the down orientation, and the rate of decrease in the capture probability as the translation away from the centre of the vestibule entrance increases, is lower than observed for the up orientation.

We also note that unlike OccD1 [17] or OprP [24] the orientation of the molecule, beyond release, does not have a noticeable impact on entry into the vestibule entrance or the translocation process, as the protein freely interacts with the various moieties on the substrate, e.g., the nucleobase, the hydroxyl group on the sugar and the phosphate group.

**Figure 3:**
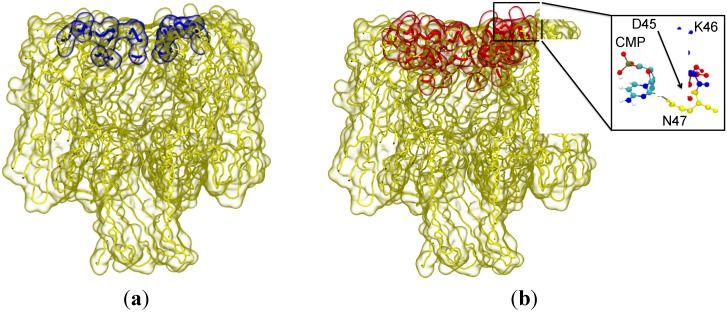
The residues with the highest propensity to interact with the nucleotide for: failed capture (**a**) and possible capture (**b**). The blue highlighted residues corresponding to failed capture are on the edge of the vestibule entrance and on the surface of the vestibule. The red highlighted residues associated with possible capture are predominantly on the edge of the entrance to the vestibule. (**Inset**) The most frequently observed binding mode between cytosine monophosphate (CMP) and the amino acid triplet, D45, K46 and N47. Hydrogen bonds (dashed lines) are observed between the hydroxyl group of the sugar and the side-chain of D45, as well as the nucleobase and the side-chain of N47. These hydrogen bonds are stable and are present for extended periods of time (greater than 1 ns). Residues are coloured for clarity; D45 in red K46 in blue, and N47 in yellow.

An in-house script was used with the graphics software Visual Molecular Dynamics (VMD) [25] to calculate the frequency and duration of Van der Waals contacts between protein residues and CMP. For simulations in which entry to the vestibule was observed, the CMP had extended interactions (an average of more than 5% over all simulations) with: D2, S3, K8, T9, D13 and N293 residues, which are located predominately in the vestibule. Whilst for simulations in which possible entry was observed, interactions were noted with residues A1, N6, K8, T9, G10, T11, D13, G15, S16, D17, T18, T19, V20, D45, K46 and N47, these residues predominately lie on the edge of the entrance to the protein. For those simulations where entry to the vestibule was not observed we noticed the majority simply didn’t interact with the protein at all, or when they did, it was with residues K8, I16, N17, T18, K46 and D47. This data and the location of these residues is summarized in Figure 3. Based on these observations we propose that the D45, K46 and N47 residues reduce the probability of entry to the vestibule via alternative favourable interactions with CMP outside the vestibule, on the surface of the cap region. The protein–nucleobase interactions that dominate here are largely hydrogen bonding of the amino acid side chains to the base and the sugar hydroxyl groups. The phosphate moiety is typically excluded from these interactions with the only exception being transient interactions with the side chain of K46 (for durations of less than 100 ps). An example interaction between CMP and the 3 residues is shown inset in Figure 3.

**Figure 4:**
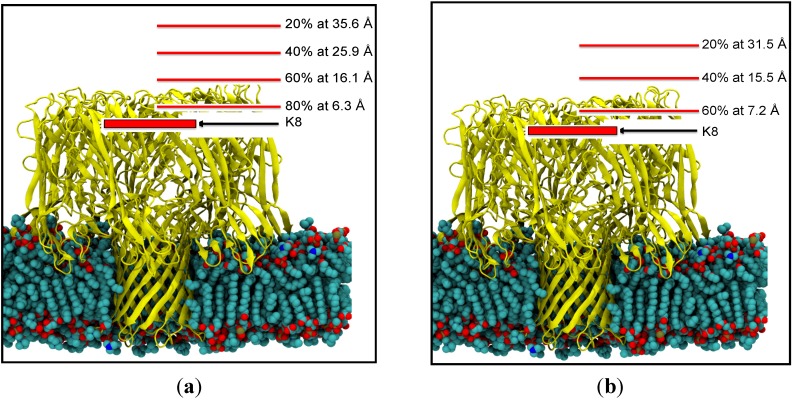
The probabilities of entry for given release points relative to the ring of K8 residues for (**a**) the phosphate down orientation and (**b**) the phosphate up orientation. Simulations performed with the phosphate in the down orientation are closer than the corresponding phosphate up simulations for the same capture probability.

Over the 360 simulations performed, translocation through the entire alpha-hemolysin protein was observed in only two simulations, as the remainder of captured CMP substrates found various binding sites in the vestibule or beta barrel. Figure 4 provides a schematic summary of the probability of entry into the vestibule as a function of distance of release.

### Sum Effects to CMP Entry into the Vestibule

Root mean squares linear regression was used to calculate the equations of the lines for the relationship between distance away from the vestibule (variable labelled “height” in equations) and probability of entry into the vestibule, shown below in Equation (1), phosphate up and Equation (2), phosphate down. It was observed that with the nucleotide at the position of K8, or zero distance, the probability of capture is 72% to 93%, demonstrating that nucleotides can diffuse rapidly under the conditions used, failing to remain captured even when released below residue N17.

(1) $$\text{Probability of possible capture}=\;72\text{\%}-(16.5\text{\% nm }{\text{height}}^{-1}\;)$$

(2) $$Probability\;of\;possible\;capture=93\text{\%}-(20.5\text{\% nm }{\text{height}}^{-1}\;)$$

The data from the simulations in which the CMP had been translated in the *xy* plane, had the effect of distance removed by setting the probability at zero translation equal to the predicted probability from the previous equations. From this data an equation was calculated using the previous method, which provides the total effect of nucleotide release location on the probability of entry into the vestibule, see Equations (3), phosphate up, and (4), phosphate down, below.

(3) $$\text{Probability of possible capture}=72\%-\left(16.5\%\;{\text{nm}}^{-1}\text{ height}\right)-(2.3\%{\text{ nm translated}}^{-1})$$

(4) $$\text{Probability of possible capture}=93\%-(20.5\%\;{\text{nm}}^{-1}\text{ height})-(6.2\%\;{\text{nm translated}}^{-1})$$

## 3. Experimental Section

Energy minimization was carried out by the method of steepest descents for either 2000 steps or a force of 1000 kJ·mol^−1^. Equilibration was using the canonical (NVT) ensemble for 1 ns, and NPT for 1 ns, using the Berendsen thermostat and barostat [26] prior to electric field equilibration for 1 ns and a field strength of 0.1 V·nm^−1^. The electric field equilibration and production molecular dynamics (MD) runs were run using the V-rescale thermostat [27] and the Parrinello-Rahman barostat [28]. All simulations were run at 310 K and 1 Bar.

The trajectories were visually analyzed using VMD to determine if the mononucleotide had drifted into the bulk water. A script was then used to compare of co-ordinates of the CMP to the c-alphas of N17, with successful capture defined as all atoms below this ring, possible capture as some above, some below and failed capture as all atoms above. The visualization step was required to prevent simulations where the nucleotide was below N17 but outside the pore being counted.

## 4. Conclusions

In conclusion, our simulations predict that optimisation of alpha-hemolysin for nanopore sequencing, which incorporates an exonuclease enzyme for cleaving nucleotides from a strand of DNA, must consider the protein-exonuclease distance of nucleotide release. We show that for successful capture of the nucleotide by the protein, the point of nucleotide release above the protein is more important than the lateral displacement of the nucleotide with respect to the dimensions of the entrance to the protein. In other words it is more important to release the nucleotide closer to the mouth of the vestibule, than it is to ensure that it is released directly above the centre of the mouth. Furthermore, our simulations reveal that the orientation of the nucleotide is also only likely to have negligible impact on the probability of entry into the protein.

Closer inspection of the wildtype alpha hemolysin revealed that residues D45, K46 and N47 play a role in interacting with CMP in instances of failed capture, therefore we recommend mutational studies to optimise this region. Of course, it must be noted that due to the number of simulations appropriate for this study, we have been unable to vary the effects of forces on the nucleotides due to the electro-osmotic flow of ions through the pore such as have been described in [29]. For a fuller picture of nucleotide dynamics in and around nanopores, in future work we plan longer simulations to include these effects and also to study all four nucleotides.
